# Modulation of Virulence-Associated Traits in *Aspergillus fumigatus* by BET Inhibitor JQ1

**DOI:** 10.3390/microorganisms10112292

**Published:** 2022-11-18

**Authors:** Anastasia Orekhova, Marta De Angelis, Andrea Cacciotti, Massimo Reverberi, Dante Rotili, Alessandra Giorgi, Virginia Protto, Graziana Bonincontro, Francesco Fiorentino, Victor Zgoda, Antonello Mai, Anna Teresa Palamara, Giovanna Simonetti

**Affiliations:** 1 Department of Public Health and Infectious Diseases, “Sapienza” University of Rome, 00185 Rome, Italy; 2 Department of Environmental Biology, “Sapienza” University of Rome, P. le Aldo Moro 5, 00185 Rome, Italy; 3 Department of Drug Chemistry and Technologies, “Sapienza” University of Rome, 00185 Rome, Italy; 4 Department of Biochemical Sciences, “Sapienza” University of Rome, 00185 Rome, Italy; 5 Institute of Biomedical Chemistry, Moscow, 10 Pogodinskaya Street, Moscow 119121, Russia; 6 Department of Infectious Diseases, Istituto Superiore di Sanità, 00161 Rome, Italy

**Keywords:** *Aspergillus fumigatus*, BET inhibitor, JQ1, extracellular proteins, Abr2, *Galleria mellonella*

## Abstract

*Aspergillus fumigatus* is a disease-causing, opportunistic fungus that can establish infection due to its capacity to respond to a wide range of environmental conditions. Secreted proteins and metabolites, which play a critical role in fungal–host interactions and pathogenesis, are modulated by epigenetic players, such as bromodomain and extraterminal domain (BET) proteins. In this study, we evaluated the in vitro and in vivo capability of the BET inhibitor JQ1 to modulate the extracellular proteins and virulence of *A. fumigatus.* The abundance of 25 of the 76 extracellular proteins identified through LC-MS/MS proteomic analysis changed following JQ1 treatment. Among them, a ribonuclease, a chitinase, and a superoxide dismutase were dramatically downregulated. Moreover, the proteomic analysis of *A. fumigatus* intracellular proteins indicated that Abr2, an intracellular laccase involved in the last step of melanin synthesis, was absent in the JQ1-treated group. To investigate at which level this downregulation occurred and considering the ability of JQ1 to modulate gene expression we checked the level of *ABR2*, *Chitinase*, and *Superoxide dismutase* mRNA expression by qRT-PCR. Finally, the capacity of JQ1 to reduce the virulence of *A. fumigatus* has been proved using *Galleria mellonella* larvae, which are an in vivo model to evaluate fungal virulence. Overall, the promising activity exhibited by JQ1 suggests that *A. fumigatus* is sensitive to BET inhibition and BET proteins may be a viable target for developing antifungal agents.

## 1. Introduction

*Aspergillus fumigatus* is an opportunistic pathogenic fungus causing high mortality diseases in immunosuppressed patients [1]. It is estimated that more than 3 million people have *A. fumigatus* chronic or invasive infections leading to over 600,000 deaths each year, with a mortality rate of 25–90% [2]. Moreover, patients with pulmonary infections that require intensive care unit management, such as those suffering from coronavirus disease 2019 (COVID-19), are at risk for secondary infections, including invasive pulmonary aspergillosis [3]. Unfortunately, *A. fumigatus* infections caused by strains resistant to antifungal drugs are increasing and this often leads to therapeutic failures. Some authors have identified azole-resistant isolates from both environmental and clinical sources. These studies have confirmed that the resistance of Aspergillus strains, which cause human infection, can be acquired from the environment [4,5].

*A. fumigatus* adapts to different environmental conditions by modulating the expression and secretion of virulence and resistance factors [6]. Secreted proteins and metabolites play a critical role in fungal–host interactions and pathogenesis. The proteases of *A. fumigatus* damage and degrade host tissue to facilitate the acquisition of essential nutrients which are required for its metabolism and promoting the penetration of hyphae into the host tissues [7]. Metabolites, such as melanin, are involved in the adhesion to host cells and resistance to phagocytosis [7,8]. Hoda et al. demonstrated the ability of melanin to prevent the intracellular killing of conidia by inhibiting the acidification of phagolysosome containing conidia [9]. Other authors indicated that 8-dihydroxynaphthalene (DHN)-derived melanin (DHN melanin) interferes with the host phagocytes, allowing the fungus to generate an environment that enabled its survival [8]. The expression of proteins and metabolites involved in virulence and resistance are controlled by epigenetic players, such as bromodomain and extraterminal domain (BET) proteins [10]. BET proteins bind to acetylated lysine residues of histones and are known to interfere with transcriptional initiation and with elongation, which, in the context of cancer, determines the inhibition of cell growth [11]. BET fungal proteins are comprehensive transcriptional regulators that control the transcription of >500 genes. [12]. The inhibition of BET proteins suppressed inflammation caused by the pathogenic fungus *A. fumigatus* in vitro [10]. Among BET inhibitors, JQ1 is a major modulator of fungal pathogen-induced inflammation [13]. JQ1 inhibits production of proinflammatory cytokines and the induction of trained immunity [14].

Here, we investigate the activity of the BET inhibitor JQ1 on virulence of the human pathogenic fungus in vitro as well as in vivo in the *G. mellonella* infection model.

## 2. Materials and Methods

### 2.1. A. fumigatus Strain and Culture Conditions

In this study, *A. fumigatus* DSM 790 (German Collection of Microorganisms, DSMZ, Braunschweig, Germany) was utilized. *A. fumigatus* was grown for 5 days on potato dextrose agar (PDA) (Sigma Aldrich, St. Louis, MO, USA). The conidia of *A. fumigatus* were then collected with phosphate-buffered saline (PBS). The inoculum consisting of *A. fumigatus* conidia was determined by spectrophotometric reading (Ultrospec™ 2100 pro) at a wavelength of 530 nm and was confirmed with the reading at the Bürker chamber. Tween 20 (Sigma-Aldrich, St. Louis, MO, USA) was not used for the preparation of the inoculum to avoid possible interference. For protein extraction, *A. fumigatus* was grown in *Aspergillus* minimal medium (MM) (1% glucose, 0.092% ammonium tartrate, 0.052% KCl, 0.052% MgSO_4_·7H_2_O, 0.152% KH_2_PO_4_, 1 mL of trace elements solution/liter [pH 6.8]) [15].

### 2.2. Antifungal Susceptibility Testing

JQ1 was synthesized as previously reported [16]. The minimum inhibitory concentration (MIC) of JQ1 against *A. fumigatus* DSM 790 was determined according to the standardized method (CLSI M38-A2 document) [17]. Initially, the molecules were solubilized in DMSO at concentrations 100 times the final concentration. This solution was then diluted 100 times with sterile RPMI and after added to the wells. The concentrations of JQ1 ranged from 6.25 µM to 100 µM. Molecule-free control, with and without 1% DMSO, was also added. *A. fumigatus* DSM 790 was grown for 120 h on PDA (Sigma-Aldrich, St. Louis, MO, USA). The concentration of the inoculum was 1.0 × 10^4^–2.5 × 10^4^ cells/mL. After 48 h, the MIC and the percent of growth reduction were calculated. All experiments were carried out, in triplicate, at least three times on separate dates.

### 2.3. JQ1 Activity against Biofilm Formation and Mature Biofilm

The anti-biofilm activity was assessed in 96-well plates as described previously [18]. *A. fumigatus* DSM 790 was grown on PDA for 5 days. The conidia were collected with PBS and counted with a hemocytometer. The inoculum concentration was 1.0 × 10^6^ cells/mL. Initially, the molecules were solubilized in DMSO at concentrations 100 times the final concentration. This solution was then diluted 100 times with sterile RPMI and after added to the wells. The concentrations in the wells of JQ1 ranged from 6.25 μM to 100 μM. Molecule-free control, with and without 1% DMSO, was added, and were incubated statically in 96-well plates at 37 °C for 48 h. To perform biofilm experiments, after 24h, the RPMI was discarded, and fresh medium containing JQ1 compounds at a concentration ranging from 6.25 µM to 100 µM, was added. The plate was incubated at 37 °C for an additional 24 h. Biofilm was quantified by measuring the metabolic activity of cells. Metabolic activity was measured using the XTT (2,3-bis(2-methoxy-4-nitro-5-sulfophenyl)-2H-tetrazolium-5-carboxanilide monosodium salt) reduction test [18]. Each experiment was executed at least three times, in triplicate, on separate days. The inhibition was expressed as percentage and calculated using this formula: 100 − [(OD_490_ of sample well/OD_490_ of positive control) × 100].

### 2.4. Conidial Surface Hydrophobicity

The surface hydrophobicity of *A. fumigatus* conidia was evaluated using an aqueous two-phase system consisting of a 1:1 mix of a dextran 260.000 solution 17.5% (*w*/*v*) (0.9 mL) and a polyethylene glycol (PEG) 3.350 solution 14.26% (*w*/*v*) (0.9 mL) in PBS, as described previously by Pihet et al. [6]. Briefly, following the addition of the conidial suspension (0.2 mL of 10^7^ conidia/mL in PBS) to the two-aqueous phase, the obtained suspensions were gently mixed and incubated at room temperature for 1 h in order to separate the two phases. An amount of 0.100 mL of each phase was sampled and its absorbance was measured at 630 nm [8].

### 2.5. Adhesion Assays

Human bronchial epithelial (BEAS-2B) cells were cultured in RPMI 1640 (EuroClone, Italy) supplemented with 1% glutamine, and 10% fetal calf serum (FCS) (HyClone, USA) at 37 °C in 5% CO_2_. For the adhesion assay, 7.0 × 10^4^ BEAS-2B cells were seeded in 24-well plates and incubated under these conditions overnight to obtain monolayers and then was used in adhesion assays. *A. fumigatus* DSM 790 and *A. fumigatus* DSM 790 with 100 μM JQ1 were grown on PDA plates for 5 days. Then the conidia were collected and suspended in sterile physiological solution. Epithelial cells were washed twice with RPMI 1640 serum-free medium and then *A. fumigatus* conidia (10^3^/well) were added. After a 1 h of incubation at 37 °C in 5% CO_2_, the medium was removed. For the lysation of epithelial cells 1 mL of 0.025% Triton-X 100 for 10 min was used. Lysed cells were centrifuged (10,000× *g* for 5 min at 4 °C) and then the samples were plated after dilution on PDA plates. The cells were counted. All experiments were carried out with two replicates [19,20].

### 2.6. Microscopic Evaluation of Adhesion

Human bronchial epithelial (BEAS-2B) cells were seeded at 6.0 × 10^5^ in 6-well plates and incubated at 37 °C overnight obtaining monolayers. Conidia obtained from *A. fumigatus* DSM 790 grown with or without JQ1were added to epithelial cells in FCS-free medium and incubated at 37 °C for 1 h. The cells were rinsed 5× with PBS and fixed in ice-cold methanol for 20 min. Medium was then discarded, the cells were washed 5× and fixed for in ice-cold methanol (50% in PBS) again for 20 min. The cells were washed by PBS, air-dried, and observed. All experiments were carried out with two replicates [19,20].

### 2.7. Radial Growth Experiment

JQ1 was dissolved in DMSO and diluted at least 100 times in the medium. An equal concentration of DMSO was added to the control. PDA plates, with and without JQ1 at concentrations ranging from 6.25 µM to 100 µM, were inoculated centrally with 5 µL of *A. fumigatus* conidia (1 × 10^6^ conidia/mL). Then the plates were incubated for 5 days. After 72, 96, and 120 h the colony diameters were measured [21]. Plotting colony diameter against time was used. The linear phase of trend was used to calculate the growth rate (mm/day). All experiments were performed with three replicates [22].

### 2.8. Protein Extraction and Quantification

The mycelium was cultured in MM without amino acids or proteins to prevent compromise of the LC-MS/MS results. Previously, some authors reported that MM induced a transcript response similar to that induced by invasive growth in the lungs of immunocompromised mice [15]. The secretome was obtained by filtering (0.22 µm filter) the culture of *A. fumigatus*. The secretome was precipitated using acetone (1:10) at −20 °C for 2 h and centrifuged at 10,000× *g* and +4 °C for 15 min. The supernatant was discarded and the samples dried for 2 h [23]. The sample buffer (0.625 M Tris (pH 6.8) 2 mL, 10% SDS 5 mL, glycerin 2 mL, distilled water 11 mL) was added to the sample. Among intracellular proteins, mycelia were washed with PBS for the removal of extracellular proteins and further contaminants. Wet mycelial biomass (0.5 g) and acid-washed glass beads (0.5 g) were then mixed with lysis buffer (10 mL) and agitated (Mini-BeadBeater, BioSpec, USA) at 2500 rpm for 10 times (60 s followed by 60 s cooling in liquid nitrogen). The suspension was then centrifuged at 6000× *g* at 4 °C for 10 min and the supernatant was collected. To remove nucleic acid contaminants the supernatant was then mixed with 7 µL/mL of DNase/RNase/Mg mix and placed on ice for 5 min. The supernatant was further treated using cold acetone (1:10) at −20 °C for 2 h. The centrifugation of the samples was made for 15 min, at 10,000× *g* and +4 °C. After the samples were dried for 2 h. Sample solubilization buffer (0.625M Tris (pH-6.8) 2 mL, 10% SDS 5.0 mL, glycerin 2.0 mL, and distilled water 11.0 mL) was used to resuspend the precipitated proteins and stored at −20 °C for later use [24]. The sample protein concentrations were measured by bicinchoninic acid (BCA) assay according to the manufacturer’s protocol (Pierce Proteins Methods, Thermo Fisher Scientific, Waltham, MA, USA) with bovine serine albumin (BSA) solutions used as standard. The calibration curves and the sample protein concentration were measured using a NanoDrop ND-1000 cuvette-free spectrophotometer (Thermo Fisher Scientific, Waltham, MA, USA) at 562 nm [25].

### 2.9. Proteomic Profile by Liquid Chromatography-Mass Spectrometry (LC-MS/MS)

Peptides obtained by digestion with trypsin of control and JQ1 samples were analyzed by shotgun proteomics strategy, entirely carried out at the Institute of Biomedical Chemistry using the equipment of the “Human proteome” Core Facility Centre (Moscow, Russia). The experiments were performed as previously described by Eldarov and colleagues [26]. The samples have been digested by trypsin using a commercial S-trap^TM^: Rapid Universal MS Sample prep (ProtiFi, Farmingdale, NY, USA). Peptides eluted from the columns were dried under vacuum and solubilized in formic acid 0.1% [27]. Briefly, the peptides were loaded onto the Acclaim-Precolumn (Thermo Scientific, Waltham, MA, USA) and were separated with high-performance liquid chromatography (HPLC, Ultimate 3000 Nano LC System, Thermo Scientific, Rockwell, IL, USA) MS analysis was performed with a Q Exactive HF mass spectrometer (Q Exactive HF Hybrid Quadrupole-Orbitrap™ Mass spectrometer, Thermo Fisher). The obtained raw data were processed with MaxQuant (Max-Planck-Institute of Biochemistry, Martinsried, Germany, version 1.6.10.43) software with the built-in search engine Andromeda. MaxQuant analyzed all samples in one run [27,28]. Protein sequences from the complete *A. fumigatus* proteome were obtained from Uniprot (August 2021) and used for protein identification with Andromeda.

### 2.10. RNA Extraction and cDNA Production

The samples were freeze-dried for one day and disrupted with mortar, pestle, and liquid nitrogen. An amount 50 mg of each mycelium (in biological duplicate) was used as starting quantity for RNA extraction with TRIzol^TM^ (Invitrogen, Waltham, MA, USA) reagent. After checking RNA integrity on 1.2 (*w*/*v*) agarose gel, we proceeded with DNA removal trough TURBO DNA—free kit (Invitrogen, Waltham, MA, USA). For cDNA synthesis, a leveling of RNA quantity was necessary (5 µg in 10 µL) optimized also for a first normalization of the following expressions analysis. The retro transcription was made with SuperScript IV (Invitrogen, Waltham, MA, USA) and random hexamers according to the manufacture’s instructions [29].

### 2.11. Quantitative Real Time-PCR (qPCR) Analysis

SYBR-Green based quantitative assays on CFX Opus 96 RT-PCR (BioRad, Hercules, CA, USA) were performed in triplicate using 1 μL from cDNA prepared as described above. The analysis has been repeated twice for each biological replicate. *A. fumigatus* elongation factor was selected as the housekeeping (reference) gene, showing the most stable expression in the biological replicates. The MS/MS sequence result was blasted in order to find the corresponding mRNA. Since all protein (except for ABR2) was reported as hypothetical, a multiple alignment analysis with ClustalΩ was performed in order to check that the full sequences were the same as those provided by the LC-MS/MS. Genes specific primers are reported in Table 1 designed with Primer3web (https://primer3.ut.ee, accessed on 6 June 2022), with a melting temperature of 59 °C. The two stepPCR conditions were the following: one cycle at 98 °C for 3 min, then 40 cycles at 98 °C for 15 min and at 59 °C for 3 min. Melting curve analysis have been performed at the end to check the specificity of the amplification reactions. After validation tests, normalization to EF1 was performed using the ΔΔCT method using the “pcr” library performed on RStudio (1.3.1093). The “Ggplot2” library was used for graphing the results.

### 2.12. Infection of G. mellonella Larvae

The larvae of *G. mellonella* were stored in wood shavings in darkness before they were used. Larvae with color alteration, such as dark spots and apparent melanization, were excluded. For the experiments, larvae weighing between 0.3 and 0.4 g were selected. Preparation of the inoculum, and larval infection were performed. Larvae were injected with sterile physiological saline, and new larvae served as controls. *G. mellonella* larvae were used to evaluate the effect of *A. fumigatus* infection (4 × 10^4^ to 5 ×10^4^ conidia/larvae) without or with (114 mg/kg larvae, 57 mg/kg larvae, 28.5 mg/kg larvae) JQ1 and the effect of *A. fumigatus* infection obtained with conidia (10^4^ conidia/larvae) coming from *A. fumigatus* growth with or without JQ1 [30].

### 2.13. Toxicity of Extracellular Proteins in G. mellonella Larvae and Melanization Assay

The in vivo model for evaluating the toxicity of extracellular proteins obtained from *A. fumigatus* treated with JQ1 or not was used. Groups of 10 *G. mellonella* larvae were inoculated with extracellular proteins at concentrations of 1.26 mg/kg larvae, 0.95 mg/kg larvae, and 0.22 mg/kg larvae.

Larvae survival was observed over 5 days. The death of the larvae was monitored by the visual inspection of the color or the absence of motion after touching forceps. Each experiment was repeated at least in triplicate [30]. Moreover, the larvae melanization has been quantified. After 3 h and 24 h of from the injection, the hemolymph was collected and diluted 1:10 with IPS buffer. For samples, 96-well plates were used. As previously described, in order to quantify the melanin, the OD at 405 nm was measured [31].

### 2.14. Statistical Analysis

Data were reported as mean ± S.E.M. The *p*-value using an unpaired *t*-test was calculated. *p* values < 0.05 were considered statistically significant. Kaplan–Meier curves were displayed for *G. mellonella* survival. GraphPad Prism 8 software (GraphPad Software Inc., San Diego, CA, USA), Perseus 1.6.15.0 (Max-Planck-Institute of Biochemistry, Martinsried, Germany), and RStudio software 1.3.1093 (RStudio, Inc., Boston, MA, USA) were used for the statistical data analysis. The library used for qRT-PCR analysis is “pcr” and “ggplot2”.

## 3. Results

### 3.1. JQ1 Shows Low Activity against A. fumigatus Planktonic Growth

The influence of JQ1 on *A. fumigatus* planktonic growth was assessed. The results demonstrated that JQ1 had a low effect on the growth of *A. fumigatus* after 48 h. The reduction of growth with respect to control was less than 10% at 100 µM (data not shown).

### 3.2. JQ1 Affects the A. fumigatus Biofilm

**Figure 1:**
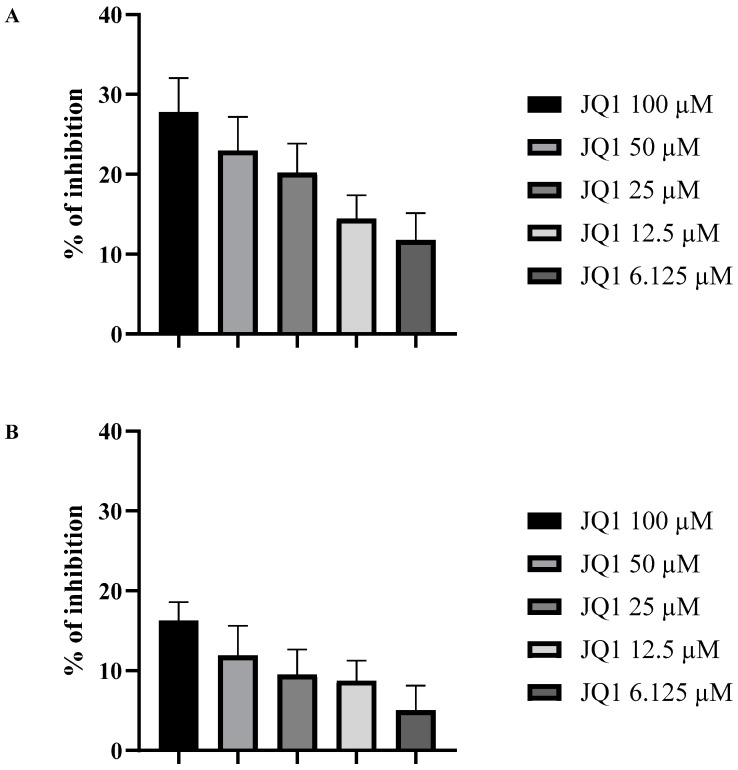
The activity of JQ1 against proliferation of *A. fumigatus* biofilm (**A**) and metabolic activity of *A. fumigatus* mature biofilm (**B**). The values are reported as the mean of three experiments performed in triplicate.

JQ1 was then assessed at a concentration range from 6.125 to 100 µM for its anti-proliferation activity towards *A. fumigatus* biofilm in 96-well plates as previously described. The results, reported in Figure 1, showed that at 100 µM, JQ1 reduced the proliferation of the biofilm by 26.6% and the mature biofilm metabolic activity of *A. fumigatus* by 17.4%.

### 3.3. JQ1 Decreases the Cell Surface Hydrophobicity

**Figure 2:**
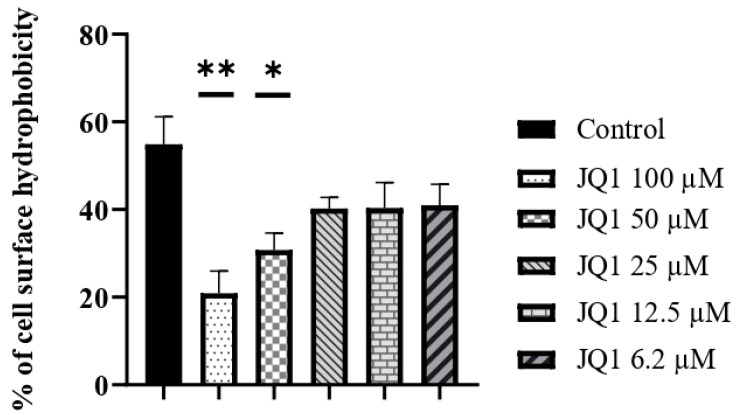
The effect of JQ1 on the surface hydrophobicity of conidia coming from *A. fumigatus* growth with or without JQ1. The values are reported as the mean of three experiments performed in triplicate. Unpaired Student *t*-tests to statistical analysis have been performed. * *p* < 0.05, ** *p* < 0.01 compared to control.

The hydrophobicity of the conidia surface of *A. fumigatus* promotes adhesion to epithelial cells [9]. *A. fumigatus* grown in the presence of JQ1 produces conidia with reduced surface hydrophobicity. In particular, the surface hydrophobicity of conidia, coming from *A. fumigatus* grown with 100 µM of JQ1, was reduced by more than 50% compared to the control conidia (Figure 2).

### 3.4. JQ1 Decreases the Cell Surface Adhesion

**Figure 3:**
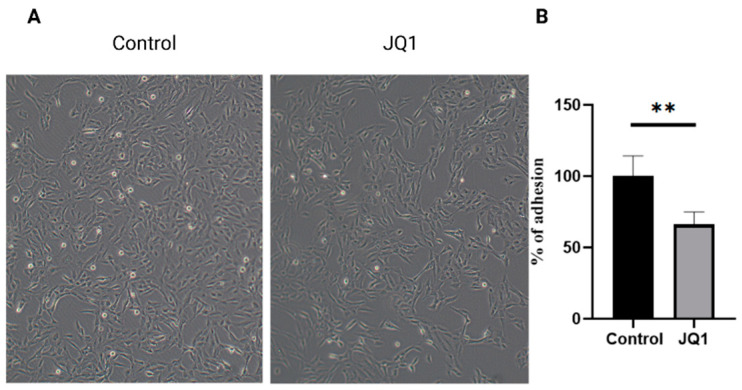
Adhesion of conidia from *A. fumigatus*, grown with or without JQ1, to human bronchial epithelial cells (BEAS-2B). Adhesion assays were performed with the conidia of *A. fumigatus.* DSM 790 and *A. fumigatus* DSM 790 treated with JQ1 100 µM (**A**). Adhesion percent to human bronchial epithelial BEAS-2B monolayers of conidia from *A. fumigatus* DSM 790 and *A. fumigatus* DSM 790 treated with JQ1 100 µM (**B**). The values are reported as the mean of three experiments. Unpaired Student *t*-tests to statistical analysis have been performed. ** *p* < 0.01 compared to the control.

The influence of JQ1 treatment on *A. fumigatus* conidia was evaluated in an in vitro model of lung infection. The adhesion on bronchial epithelial cells (BEAS-2B) of conidia obtained from *A. fumigatus* grown with or without JQ1 (100 µM) was evaluated [19,20]. Microscopic observations showed that pretreatment with JQ1 100 µM significantly reduced the adhesion to the epithelial cells of *A. fumigatus* conidia (Figure 3A). To confirm the results obtained with microscopic observation, the conidia were cultured, and the CFU were evaluated. Consistent with the microscopic observation, the number of adherent conidia, obtained from *A. fumigatus* grown with JQ1 100 µM, was significantly reduced compared to the conidia obtained from A. fumigatus without JQ1 (Figure 3B).

### 3.5. JQ1 Reduces the Radial Growth Rate of A. fumigatus

**Figure 4:**
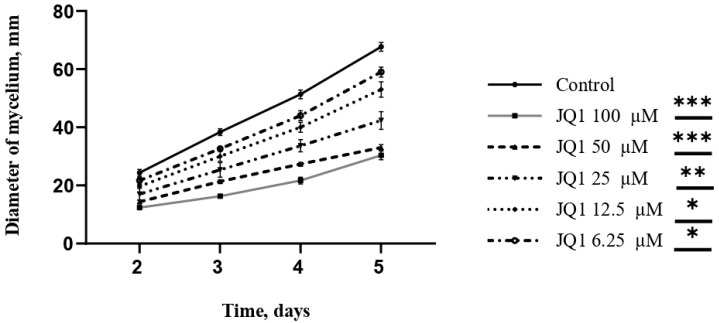
JQ1 activity on the radial growth rate of *A. fumigatus*. The colony diameter of *A. fumigatus* after 2, 3, 4, and 5 days of cultivation, with different concentrations of JQ1. The value is expressed as the media of at least three independent experiments. Unpaired Student *t*-tests to statistical analysis have been performed. * *p* < 0.05; ** *p* < 0.01; *** *p* < 0.001 compared to control.

JQ1 was then tested at concentrations ranging from 6.25 µM to 100 µM to investigate its influence on the growth of mycelium. The colony diameter at 48, 72, 96, and 120 h after incubation was measured. The results showed a reduction in growth and a change in mycelium color in a dose-dependent manner. *A. fumigatus* grown with JQ1 100 µM, after 120 h, had a colony diameter 55.2% smaller than the control (Figure 4).

### 3.6. JQ1 Changes the Concentration of Extracellular Virulence Proteins

**Figure 5:**
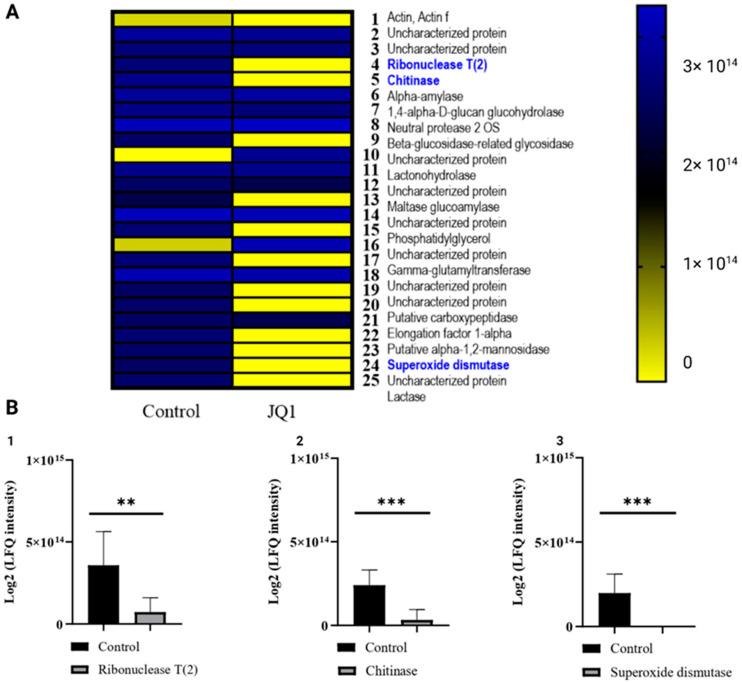
LC-MS/MS results of proteins secreted by *A. fumigatus* DSM 790 growth with JQ1 or not. (**A**) Extracellular proteins with significantly different abundance after JQ1 treatment. (**B**) Log2 (LFQ intensity) of extracellular proteins ribonuclease (1), chitinase (2), and superoxide dismutase (3) after treatment with JQ1. The values are expressed as mean of at least three biological replicates. Unpaired Student *t*-tests to statistical analysis were performed. ** *p* < 0.01, *** *p* < 0.001 compared to control.

To obtain the qualitative and quantitative compositions of proteins secreted by *A. fumigatus* in the presence or the absence of JQ1, LC-MS/MS was performed on extracellular and intracellular proteins secreted by *A. fumigatus* DSM 790. Tandem MS/MS analysis was performed for 24,358 *m/z* values and data on primary structure were obtained for thousands of peptides. The LC-MS/MS approach allowed the identification of a total of 76 proteins, of which 57 extracellular were defined for at least two peptides each (Supplementary S1). Among them, 25 proteins showed a significant difference in abundance when comparing the extracts obtained from *A. fumigatus* grown with JQ1 and *A. fumigatus* grown without JQ1 (Figure 5). In particular, virulence proteins, such as ribonuclease, chitinase II, and superoxide dismutase (Cu/Zn), resulted in under-expression or absence in the samples pretreated with 100 µM JQ1 [32,33,34,35].

### 3.7. JQ1 Reduces the Protein Related to Melanin Synthesis

**Figure 6:**
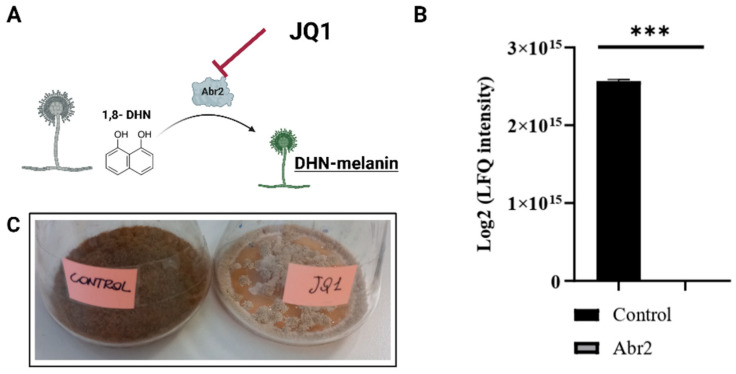
LC-MS/MS results of intracellular proteins of *A. fumigatus* DSM 790 growth with JQ1 or not. (**A**) Scheme of melanin pathway in *A. fumigatus.* (**B**) Log2 (LFQ intensity) of Abr2 intracellular fraction after treatment with JQ1. (**C**) *A. fumigates* DSM 790 growth with or without JQ1. At least three replicates were utilized for each experiment. Unpaired Student *t*-tests to statistical analysis have been performed. *** *p* < 0.001 compared to control.

Proteomic analysis also enabled the detection of 231 intracellular proteins, which were identified for at least two peptides (Supplementary S2). After treatment with JQ1, the LC-MS/MS analysis of the intracellular fraction indicated the reduced level of catalase, *D*-xylose reductase, glyoxalase II, AB hydrolase-1 domain-containing protein, phosphoglucomutase, putative formamidase, uncharacterized proteins, and the absence of the protein aspergillus brown 2 (Abr2) (Figure 6B). Some of these proteins, such as catalase and glyoxalase II, are well-known virulence factors of *A. fumigatus*, other proteins are responsible for the metabolism of fungal cells. Abr2 is involved in the last step of the melanin pathway (Figure 6A).

### 3.8. JQ1 Reduces the Gene Expression of Extracellular and Intracellular Virulence Factors

**Figure 7:**
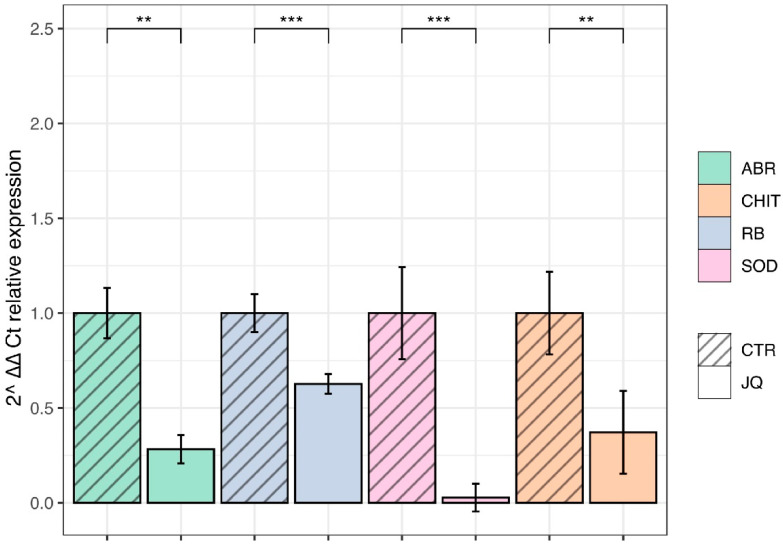
RT-PCR shows the mRNA of *A. fumigatus* expressions of *ABR2* (ABR), *Chitinase* (CHIT), *Ribonuclease* (RB), and *Superoxide dismutase* (SOD) in the control (CTR) and JQ1-treated (JQ) groups. RT-PCR results show the normalized C_T_ value of target genes after subtracting that of reference gene (*elongation factor*-1). Unpaired Student *t*-tests to statistical analysis have been performed. ** *p* < 0.01; *** *p* < 0.001 compared to control.

In order to quantify the gene expression of downregulated proteins produced by *A. fumigatus* in the presence or the absence of JQ1, such as Abr2, chitinase, ribonuclease, and superoxide dismutase, and qRT-PCR was performed. SYBR-Green-based quantitative assays were performed in triplicate with two independent biological replicates. The results showed that JQ1 significantly downregulated the mRNA levels of *ABR2* (KAH1289617.1), *Chitinase* (KAH2766398.1), *Superoxide dismutase* (KAF4252661.1), and *Ribonuclease* (KAH2174949.1) in the treated groups (*p* < 0.05) were compared to the control (Figure 7). These results suggest that *A. fumigatus* exerts an epigenetic control of the expression of *Ribonuclease*, *ABR2*, *Chitinase*, and *Superoxide dismutase* whose products are important for its virulence.

### 3.9. JQ1 Reduces the Mortality of G. mellonella Larvae Infected with A. fumigatus

**Figure 8:**
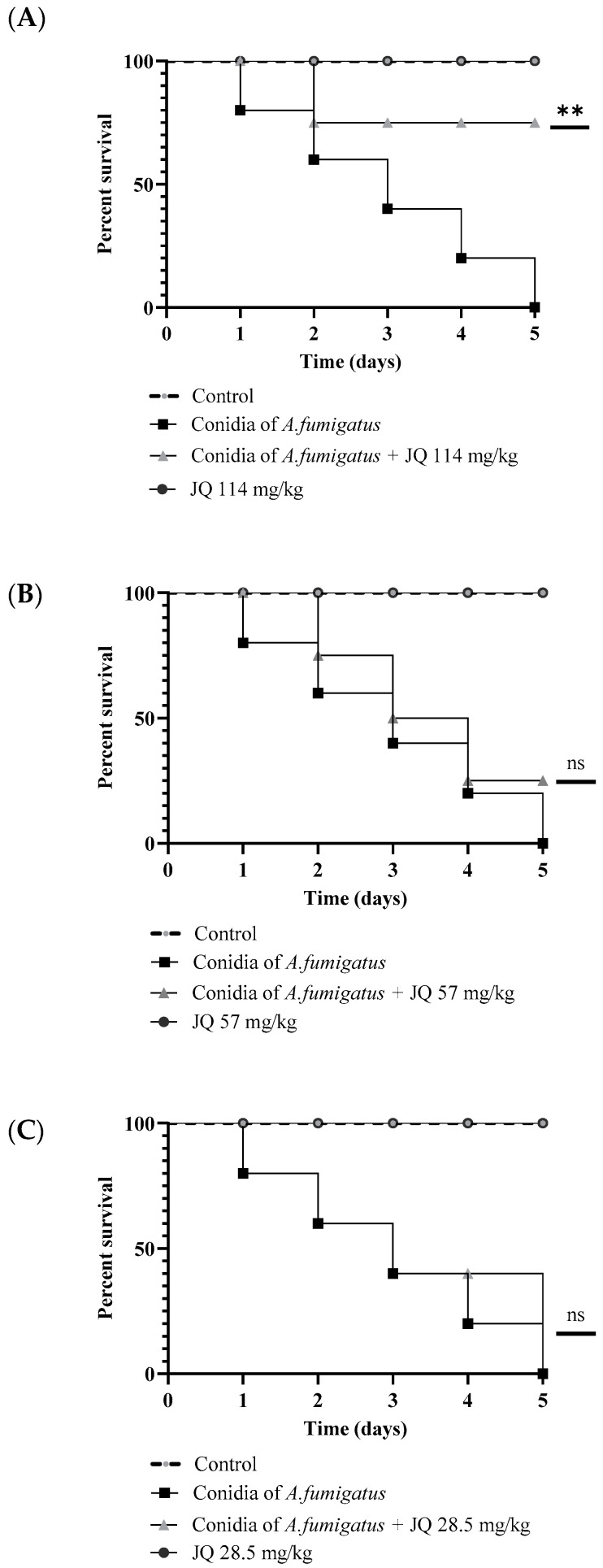
Activity of JQ1 on *G. mellonella* infected with *A. fumigatus*. *G. mellonella* survival curves. Larvae (n = 15/strain) were infected with *A. fumigatus* conidia (5 × 10^4^ conidia/larvae) and monitored daily for 5 days post-infection. JQ1 concentration 114 mg/kg (**A**), JQ1 concentration 57 mg/kg (**B**), JQ1 concentration 28.5 mg/kg (**C**). Statistical significance relative to control was assessed by Kaplan–Meier followed by Mantel–Cox log-rank test. Three experiments on different dates were performed. ** *p* < 0.01 compared to *A. fumigatus* without JQ1.

*G. mellonella* larvae are used as an in vivo model to evaluate the activity of antifungal agents. Furthermore, *G. mellonella* larvae are used to evaluate the virulence of fungi of medical interest, such as aspergillus, since the immune response of the larvae is similar to the innate response of vertebrates. In this study, *G. mellonella* was infected with *A. fumigatus* conidia and treated with different concentrations of JQ1. Mortality curves were determined to calculate the lethal dose (data not shown). The survival rate of larvae was observed daily for 5 days after inoculation with 5 × 10^4^ conidia/larvae of *A. fumigatus* with or without JQ1 Results showed that by injecting 114 mg/kg of JQ1 to larvae inoculated with *A. fumigatus*, the survival of the larvae increased compared to larvae inoculated with *A. fumigatus* only. As shown in Figure 8, survival had increased by 80% after 5 days of treatment. PBS inoculated larval groups had 0% mortality. Conversely, the mortality rate was 100% at day 5 post-infection in the group of larvae infected with *A. fumigatus* conidia. The findings reveal a clear relationship between JQ1 concentration and mortality (Figure 8).

### 3.10. Pretreatment of A. fumigatus with JQ1 Reduces the Mortality in G. mellonella Model

**Figure 9:**
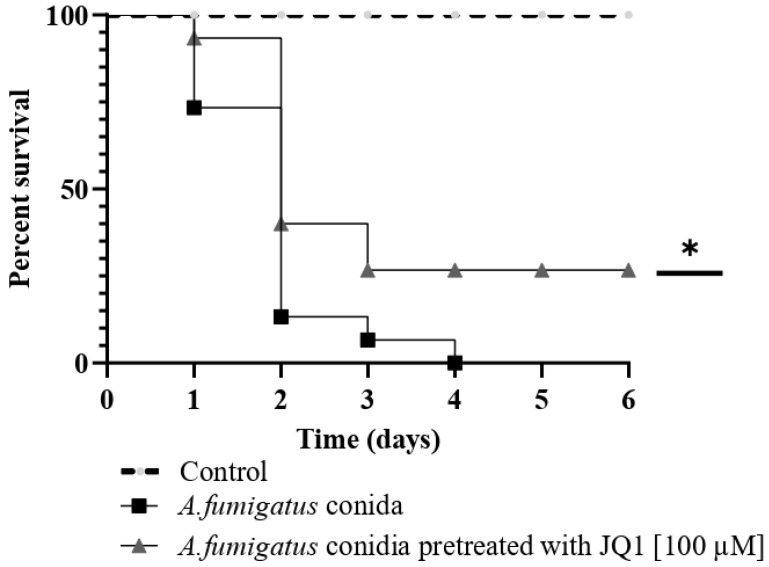
The mortality of *G. mellonella* following injection with conidia obtained from *A. fumigatus* treated with JQ1or not. *G. mellonella* survival curves. Larvae (n = 15/strain) were infected with conidia (10^4^ conidia/larvae) from *A. fumigatus* grown with or without JQ1. Larvae were monitored daily for 6 days post-infection. Statistically significance compared to control was assessed by the Kaplan–Meier followed by Mantel–Cox log-rank test. Three experiments on different dates were performed. * *p* < 0.05.

Figure 9 shows the mortality of *G. mellonella* larvae following injection with conidia coming from *A. fumigatus* growth with or without JQ1 at 100 µM. Treatment with JQ1 increases the survival of *G. mellonella* to 26.7% after 6 days post-infection.

### 3.11. The Toxicity of Extracellular Protein in G. mellonella Larvae was Reduced after Treatment of A. fumigatus with JQ1

**Figure 10:**
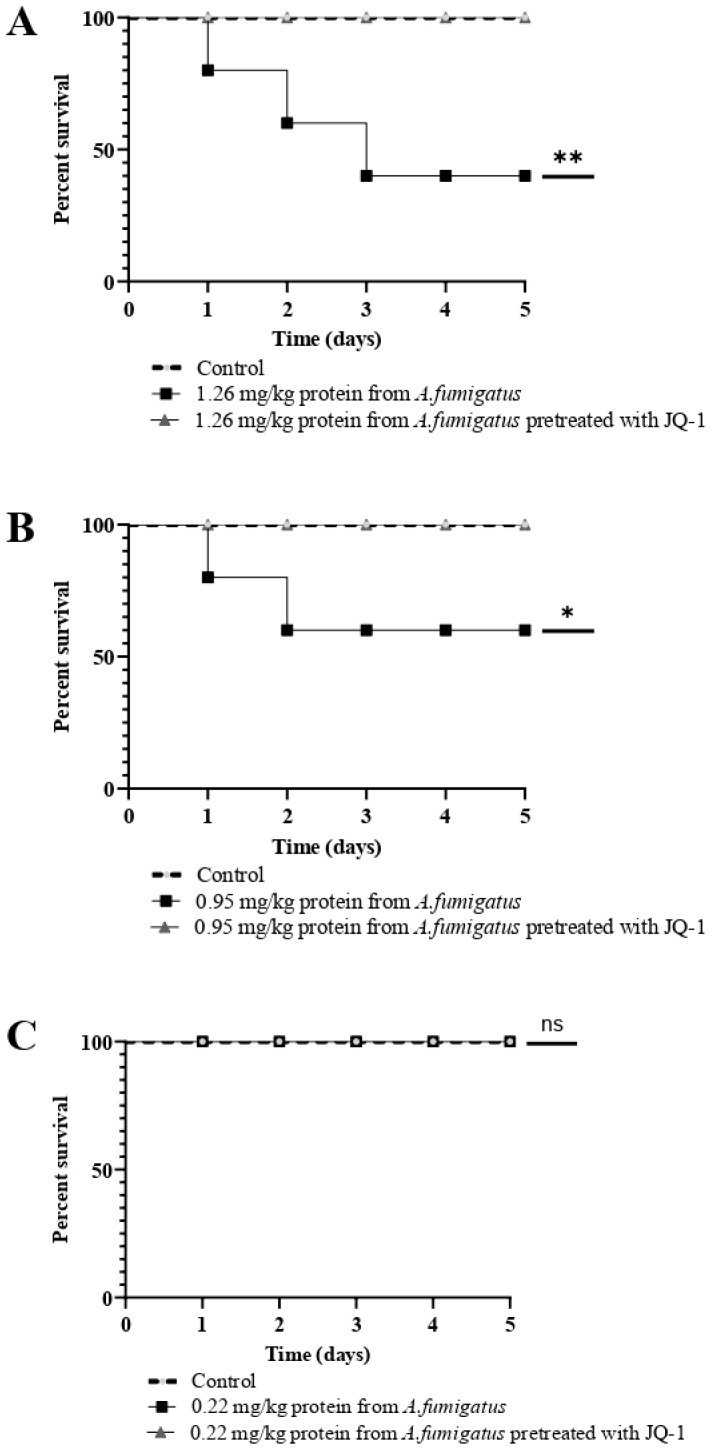
Effect of extracellular proteins obtained from *A. fumigatus* treated or not with 100 µM of JQ1. Proteins concentration 1.26 mg/kg larvae (**A**), proteins concentration 0.95 mg/kg larvae (**B**), proteins concentration 0.22 mg/kg larvae (**C**). *G. mellonella* survival curves (n = 15/strain). Larvae were checked daily for 5 days post-infection. Statistical significance compared to control was assessed by the Kaplan–Meier followed by Mantel–Cox log-rank test. Three experiments on different dates were performed. * *p* < 0.05 compared to the conidia from *A. fumigatus*; ** *p* < 0.01 compared to the conidia from *A. fumigatus*.

To investigate the role of JQ1 in the expression of *A. fumigatus* virulence proteins, extracellular proteins (26 mg/kg larvae, 0.95 mg/kg larvae, and 0.22 mg/kg larvae) of obtained from *A. fumigatus* cultured with or without 100 µM JQ1 were injected into *G. mellonella* larvae. Figure 10 shows *G. mellonella* survival curves after the injection of *A. fumigatus* extracellular proteins. Notably, the pretreatment of *A. fumigatus* with JQ1 significantly decreased the mortality rate of *G. mellonella* caused by secreted protein extracts in a dose-dependent manner [36,37].

**Figure 11:**
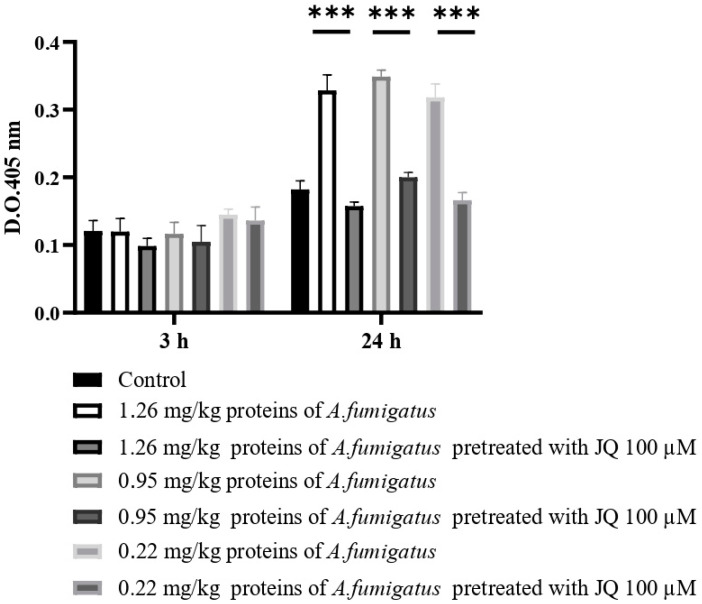
The melanization of larvae hemolymph after the injection of different concentrations of proteins from *A. fumigatus* and JQ1. At least three independent experiments were performed. Unpaired Student *t*-tests to statistical analysis were performed. *** *p* < 0.001 compared to the conidia from *A. fumigatus*.

The activity of extracellular proteins obtained from *A. fumigatus* pretreated or not with JQ1 on the larvae melanization was evaluated. Proteins ranging from 1.26 mg/kg larvae to 0.22 mg/kg larvae were injected in *G. mellonella* larvae. After 24 h, the melanization level was assessed. These results showed that there was a significant difference (*** *p* < 0.001 compared to the control) between the values obtained from the proteins of *A. fumigatus* pretreated and untreated with JQ1 (Figure 11).

## 4. Discussion

Triazole treatment is the most common therapeutic approach for diseases caused by *A. fumigatus* [38]. However, *A. fumigatus* strains resistant to azoles have now increased, representing a significant problem [35,38,39]. The increase in azole-resistant strains is also due to the widespread use that has been made of azole-based treatments to combat fungal infections in food plants [40]. Consequently, the availability of effective antifungal drugs is very limited due to development of global resistance to currently available antifungal agents [41].

In this study, the activity on the *A. fumigatus* virulence of JQ1, a thienotriazolodiazepine derivative, was evaluated. JQ1 is a epigenetic modulator, inhibiting the human BET family proteins BRD2, BRD3, BRD4, and BRDT, with a preference for BRD4 [16]. Some authors have reported the anti-cancer effects of JQ1, including the inhibition of proliferation and the promotion of apoptosis in various cancer types, both solid [42,43,44] and hematological [11,45].

In this study, the decrease in *Aspergillus* virulence after treatment with JQ1 was demonstrated. Indeed, extracellular ribonuclease, chitinase II, and superoxide mutase (Cu/Zn) were significantly reduced after the treatment of *A. fumigatus* with JQ1.

Superoxide dismutase represents a cellular antioxidant defense system by detoxifying oxygen radicals and a virulence factor of *Aspergillus*. Indeed, previous reports indicated that the deletion mutants of superoxide dismutase display reduced virulence [34,46]. Ribonuclease toxins kill cells by inhibiting protein synthesis through the hydrolysis of the phosphodiester bond of 28S rRNA [47]. These toxins are critical virulence factors of *A. fumigatus* [48]. Ribotoxins are the major IgE-binding allergens implicated in allergic bronchopulmonary aspergillosis, aspergilloma, and cystic fibrosis with the complication of allergic bronchopulmonary aspergillosis [32]. The chitinases in filamentous fungi are present in the extracellular matrix, in the hyphal branching sites, and in the germ tube. [33]. Chitinases may be involved in biofilm maturation. Biochemical and transcriptional analyses showed that chitinase inhibition affected the growth of the biofilm and the stability of mature biofilm. The mRNA levels of chitinase are significantly upregulated in the biofilm. Consistently, acetazolamide, a weak fungal chitinase inhibitor (AfChiA1 IC_50_ > 150 μM), reduces *A. fumigatus* biofilm biomass [49]. Previous studies have suggested the clinical usage of chitinase inhibitors as anti-biofilm agents [42]. In the present study, a 55.2% decrease in *A. fumigatus* radial growth after exposure to 100 µM of JQ1 was observed. Moreover, *A. fumigatus* treatment with JQ1 at the same concentration led to a 26.6% reduction in biofilm formation and a 17.4% reduction in mature biofilm.

In the early stages of infection, the conidia must defend themselves from the host immune responses and adhere to epithelial cells. *A. fumigatus* conidia contain melanin to defend against oxidative stress from reactive oxygen species and alveolar macrophages killing [50]. To this end, DHN-melanin interferes with the host phagocytes, with therapy enabling the fungus to generate surroundings that facilitate its survival. This occurs via a two-step mechanism: firstly, phagocytosed melanized conidia inhibit phagolysosome acidification, thus reducing the killing of fungi by macrophages; secondly, while residing in non-acidified phagolysosomes, conidia inhibit the apoptosis of the macrophages, thus providing an intracellular niche for conidial survival. Some authors have reported that melanin has a further role in virulence as it is necessary for the assembly of the conidia wall [8]. Furthermore, previous studies showed that Abr2 knockout conidia did not show virulence in the in vivo infection model [51]. In other experiments, *A. fumigatus* conidia with Abr2 mutation reduced the level of phagolysosome acidification [7]. In the present study, proteomics experiments indicated the absence in the intracellular fraction of the protein Abr2. Moreover, a significative reduction of *ABR2* mRNA levels when using qRT-PCR to quantify gene expression was demonstrated.

Abr2 is involved in the last step of the DHN-melanin formation pathway and modulates conidia pigmentation [52]. Thus, the changed colour of conidia after treatment with JQ1 could be related to Abr2 inhibition. Moreover, it was reported that melanin increases the negative charge and the hydrophobia of the conidia [9], and other authors reported that there is a direct relationship between adherence to the host surface and cell surface hydrophobicity [9,53]. Consistently, our experiment showed a decrease in the cell surface hydrophobicity of conidia from JQ1-treated *A. fumigatus* compared to the control, suggesting that JQ1 blocked the melanin biosynthetic pathway and reduced certain hydrophobic components on the conidial surface.

The modulation of fungal virulence has been investigated by many authors using *G. mellonella* larvae as its immune response presents similarities with the innate vertebrate immune response [54]. To evaluate *A. fumigatus* virulence after treatment with JQ1, in vivo experiments using *G. mellonella* showed that the extracellular proteins obtained after treatment of *A. fumigatus* with 100 µM JQ1 were less virulent and reduced the *G. mellonella* mortality. Additionally, larvae treatment with JQ1 reduced the *G. mellonella* mortality after *Aspergillus* conidia injection. To evaluate the activity of JQ1 on *A. fumigatus* conidia, the strain was grown with JQ1 and the conidia were inoculated in *G. mellonella* larvae. The results showed reduced mortality and a reduced melanization of larvae. It is well known that the melanization of hemolymph is the early humoral reaction of larvae and occurs rapidly after infection [31].

Melanin formation in *G. mellonella* is catalyzed by phenoloxidase [55]. It is well known that phenoloxidase enzyme activity is a marker for immune response in *G. mellonella* [56]. Indeed, the activation of phenoloxidase occurs after the recognition of non-self-components [57] and humoral factors, such as enzymes controlling the activity of phenoloxidase influence melanin synthesis. Khan et al. showed that the injection of extracellular proteins from the entomopathogenic fungus initiated the melanization of larvae due to converting inactive pro-phenoloxidase to active phenoloxidase [58].

The results of this study show that there is a significant difference between the number of proteins of JQ1-treated and untreated *A. fumigatus*. *A. fumigatus* controls the expression of some of its virulence factors, such as *Abr*, *Chit*, and *Sod* by chromatin reshaping. Other species belonging to the *Aspergillus* genus, such as *Aspergillus flavi*, control the expression of secondary metabolism and virulence similarly [59,60]. Therefore, using epigenetic modulators for switching off the virulence genes could pave the way for identifying new targets for the development of antifungals to fight infections without inducing resistance. Indeed, the use of epigenetic modulators that reduce virulence without killing the microorganism could be a winning strategy as it may prevent the development of resistance. Given the promising efficacy of JQ1, it would be interesting to develop fungal BET specific inhibitors to further support the hypothesis that fungal BET inhibition may be a viable approach for developing new antifungal agents.

## Figures and Tables

**Table 1:** Nucleotide sequence of *A. fumigatus* primers for RT-PCR.

Gene Name	Primer	Nucleotide Sequence	GenBank
AFUA_1G06390 (*Elongation factor*-1)	EF1-F	5′-AGGTCATCGTCCTCAACCAC-3′	XM_745295.2
	EF1-R	5′-ACCGGACTTGATGAACTTGG-3′	
AFUA_2G17530 (*Conidial pigment biosynthesis oxidase ABR2*)	ABR-F	5′-CAATCAAAGAGGCCAAGGAG-3′	KAH1289617.1
	ABR-R	5′-TATGGCAGTGCAACAGGAAC-3′	
AFUA_1G11640 (*Superoxide dismutase putative*)	SOD-F	5′-TCTCCCACATAGACAGAACACG-3′	KAF4252661.1
	SOD-R	5′-ATGCTAGGGCTTCATTGTCG-3′	
AFUA_1G16600 (*Ribonuclease T2 putative*)	RB-F	5′-GCTGCTGTCCTACATGCAAA-3′	KAH2174949.1
	RB-R	5′-GGCCCTTGAAAAGATCAACA-3′	
AFUA_3G11280 (*Chitinase class V chitinase*, *putative*)	CHIT-F	5′-TTTACGGCTGCATCAAACAG-3′	KAH2766398.1
	CHIT-R	5′-GAGCCTCAACTTCGTTCTGG-3′	

## Data Availability

Data are available upon request.

## Supplementary Materials

The following supporting information can be downloaded at: https://www.mdpi.com/article/10.3390/microorganisms10112292/s1. Supplementary S1. LC-MS/MS results of proteins secreted by *A. fumigatus* DSM 790 after or not after JQ1 treatment. Overall data of LC-MS/MS analysis after treatment of *A. fumigatus* with or without JQ1. Log2 (LFQ intensity) of the virulence proteins. The value is expressed as media of at least three independent biological replicates in triplicate. Supplementary S2. LC-MS/MS results of intracellular proteins by *A. fumigatus* DSM 790 after or not after JQ1 treatment. Overall data of LC-MS/MS analysis after treatment of *A. fumigatus* with or without JQ1. Log2 (LFQ intensity) of the virulence proteins. The value is expressed as media of at least three independent biological replicates.
