# Supplementary material for: Modulation of Virulence-Associated Traits in Aspergillus fumigatus by BET Inhibitor JQ1
Source: Microorganisms. 2022 Nov 18;10(11):2292. doi: 10.3390/microorganisms10112292 (PMC9698166; doi:10.3390/microorganisms10112292)
Supplement: Supplementary file 1 [file microorganisms-10-02292-s001.zip › Supplementary S2.pdf]

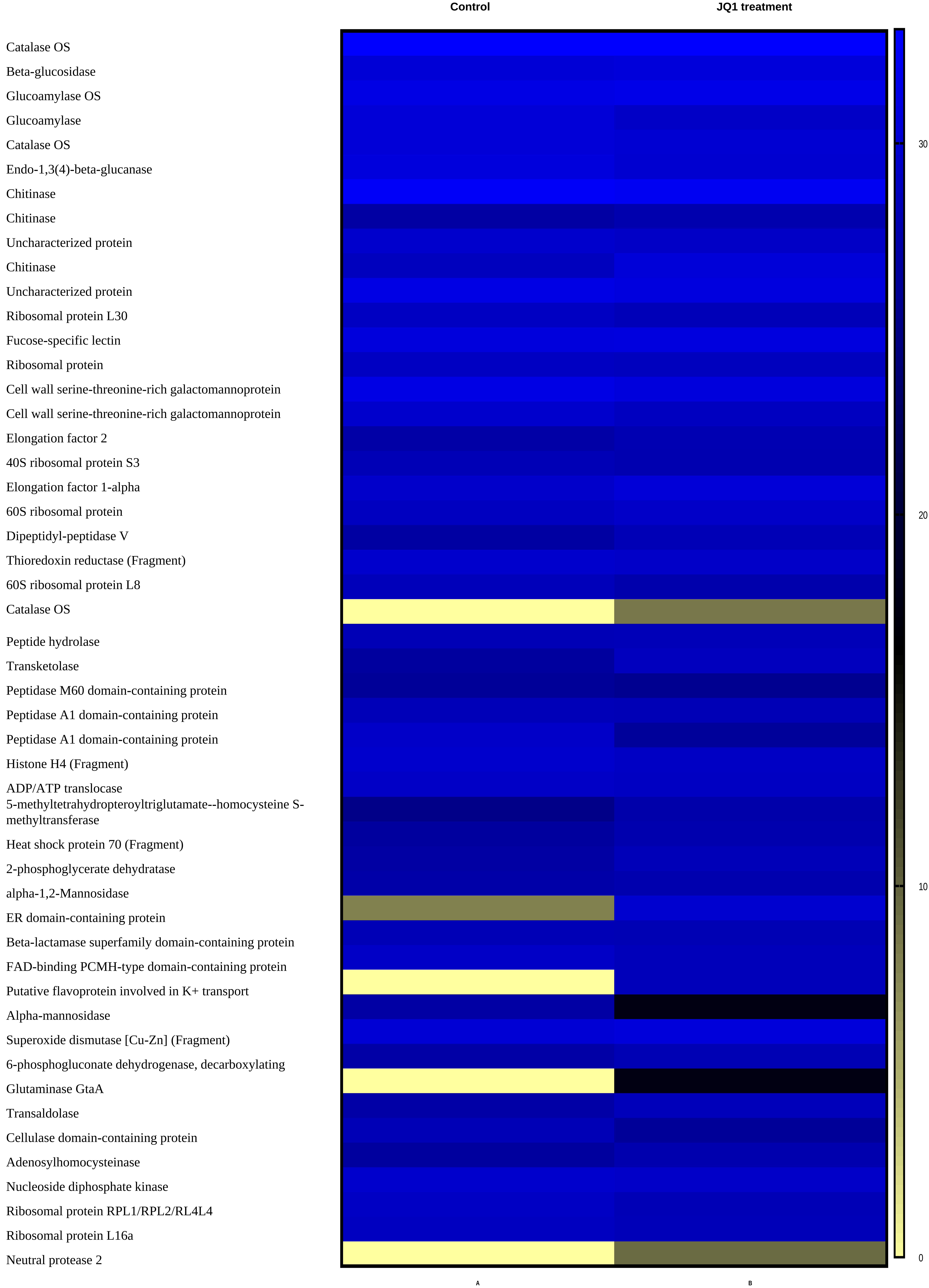

Supplementary S2. LC-MS/MS results of intracellular proteins by *A. fumigatus* DSM 790 after or not after JQ1 treatment. Overall data of LC-MS/MS analysis after treatment of *A. fumigatus* with or without JQ1. Log 2 (LFQ intensity) of the intracellular proteins. The value is expressed as media of at least three independent biological replicates.

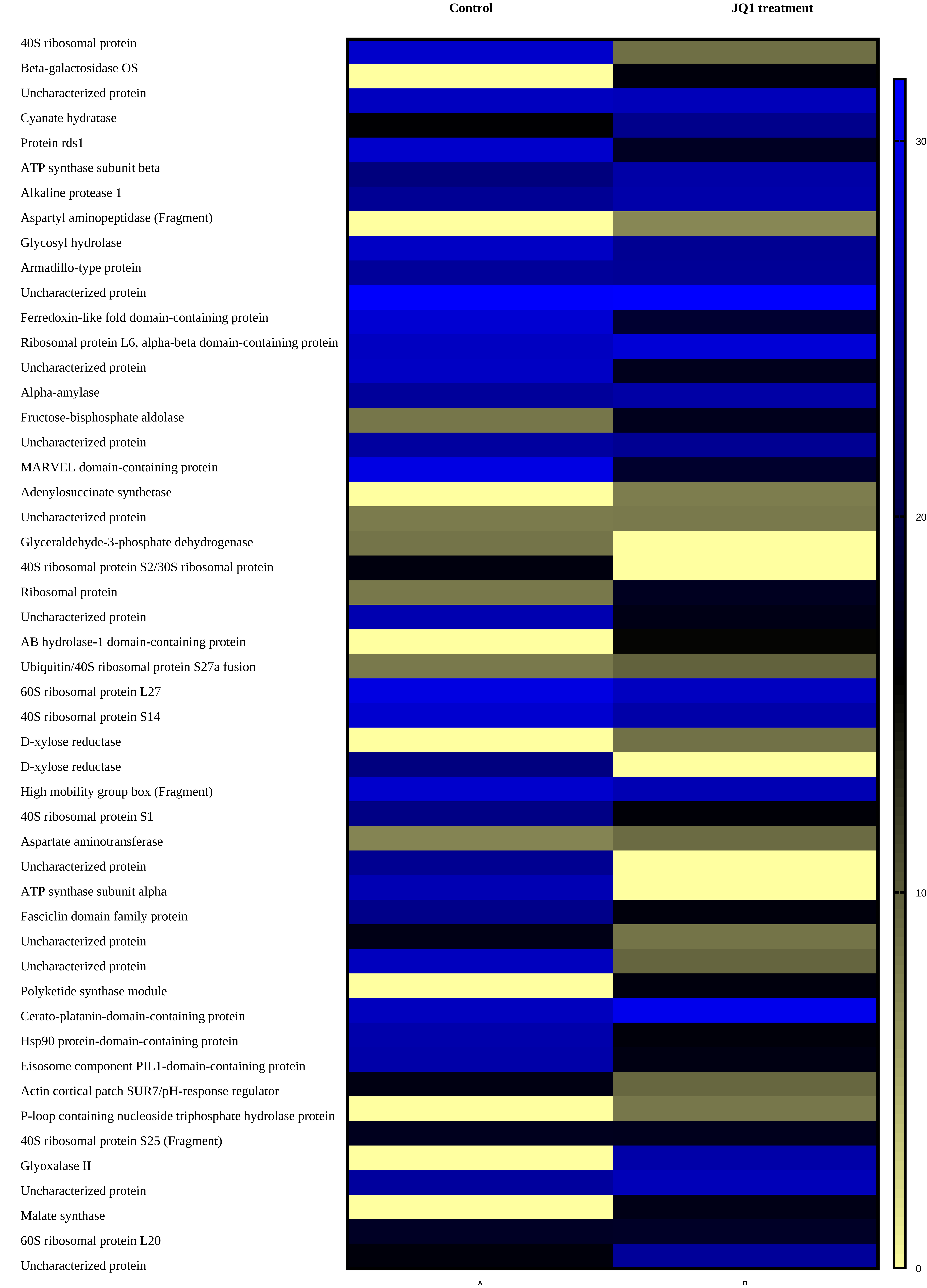

Supplementary S2. LC-MS/MS results of intracellular proteins by *A. fumigatus* DSM 790 after or not after JQ1 treatment. Overall data of LC-MS/MS analysis after treatment of *A. fumigatus* with or without JQ1. Log 2 (LFQ intensity) of the intracellular proteins. The value is expressed as media of at least three independent biological replicates.

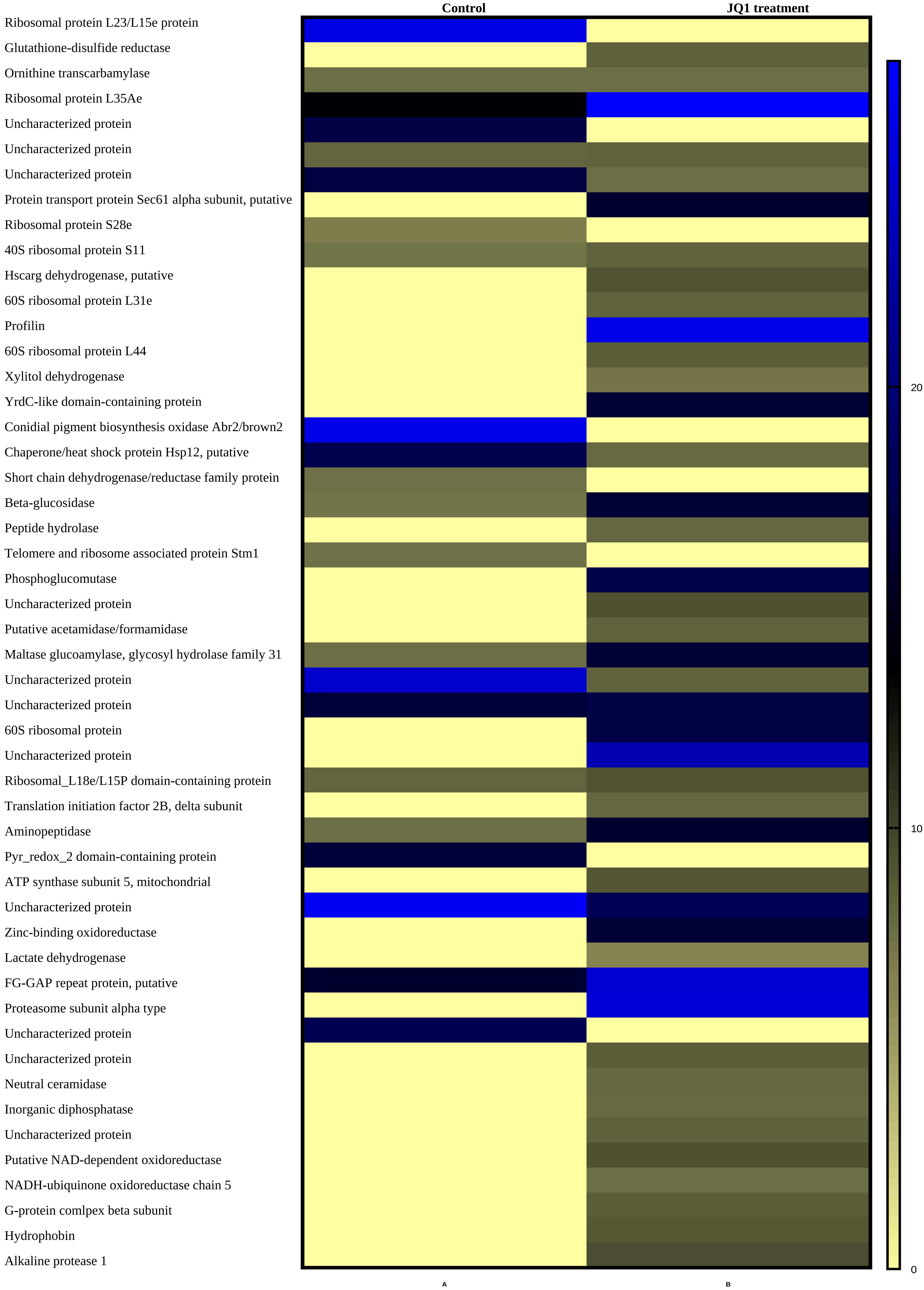

Supplementary S2. LC-MS/MS results of intracellular proteins by *A. fumigatus* DSM 790 after or not after JQ1 treatment. Overall data of LC-MS/MS analysis after treatment of *A. fumigatus* with or without JQ1. Log 2 (LFQ intensity) of the intracellular proteins. The value is expressed as media of at least three independent biological replicates.

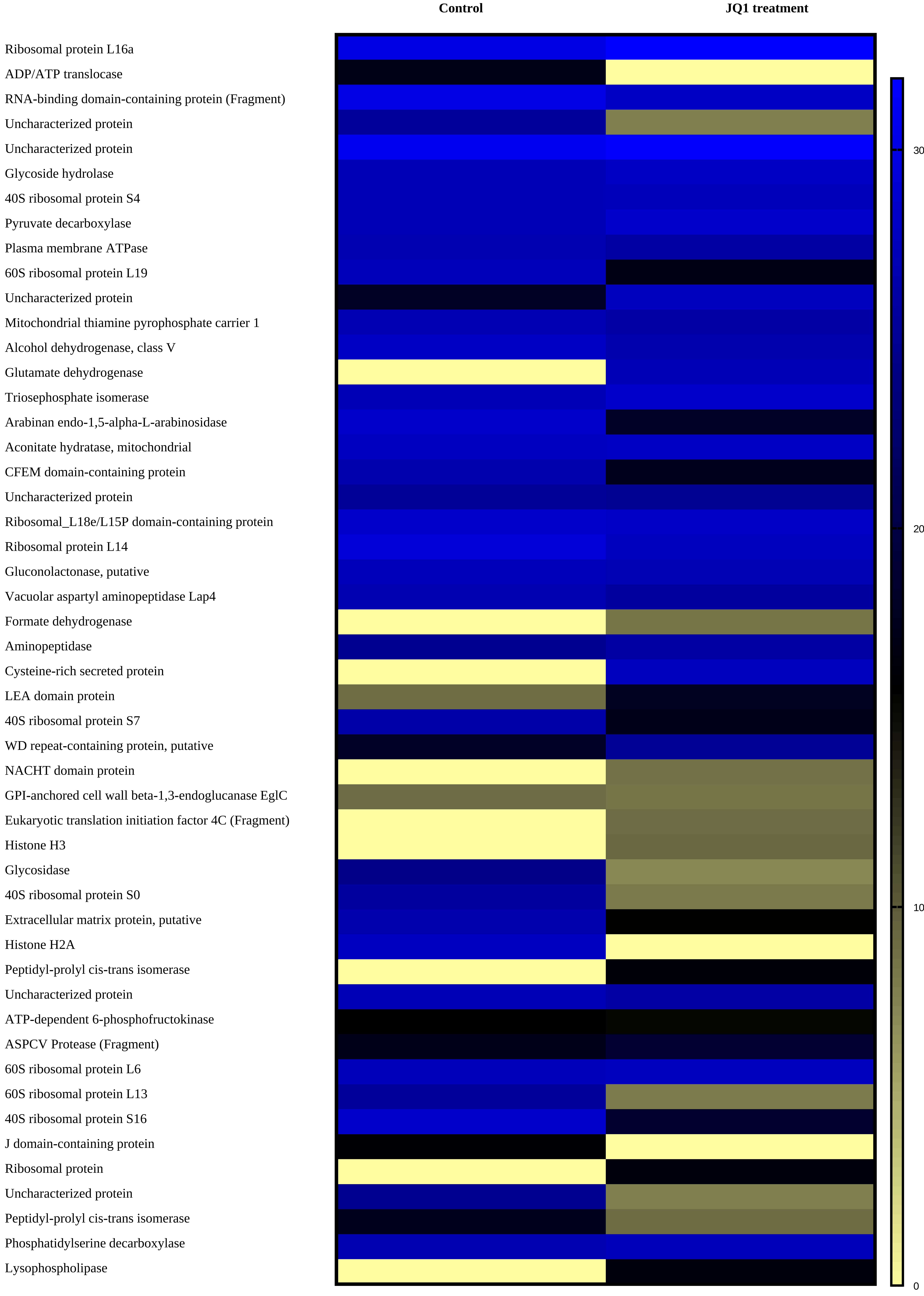

Supplementary S2. LC-MS/MS results of intracellular proteins by *A. fumigatus* DSM 790 after or not after JQ1 treatment. Overall data of LC-MS/MS analysis after treatment of *A. fumigatus* with or without JQ1. Log 2 (LFQ intensity) of the intracellular proteins. The value is expressed as media of at least three independent biological replicates.

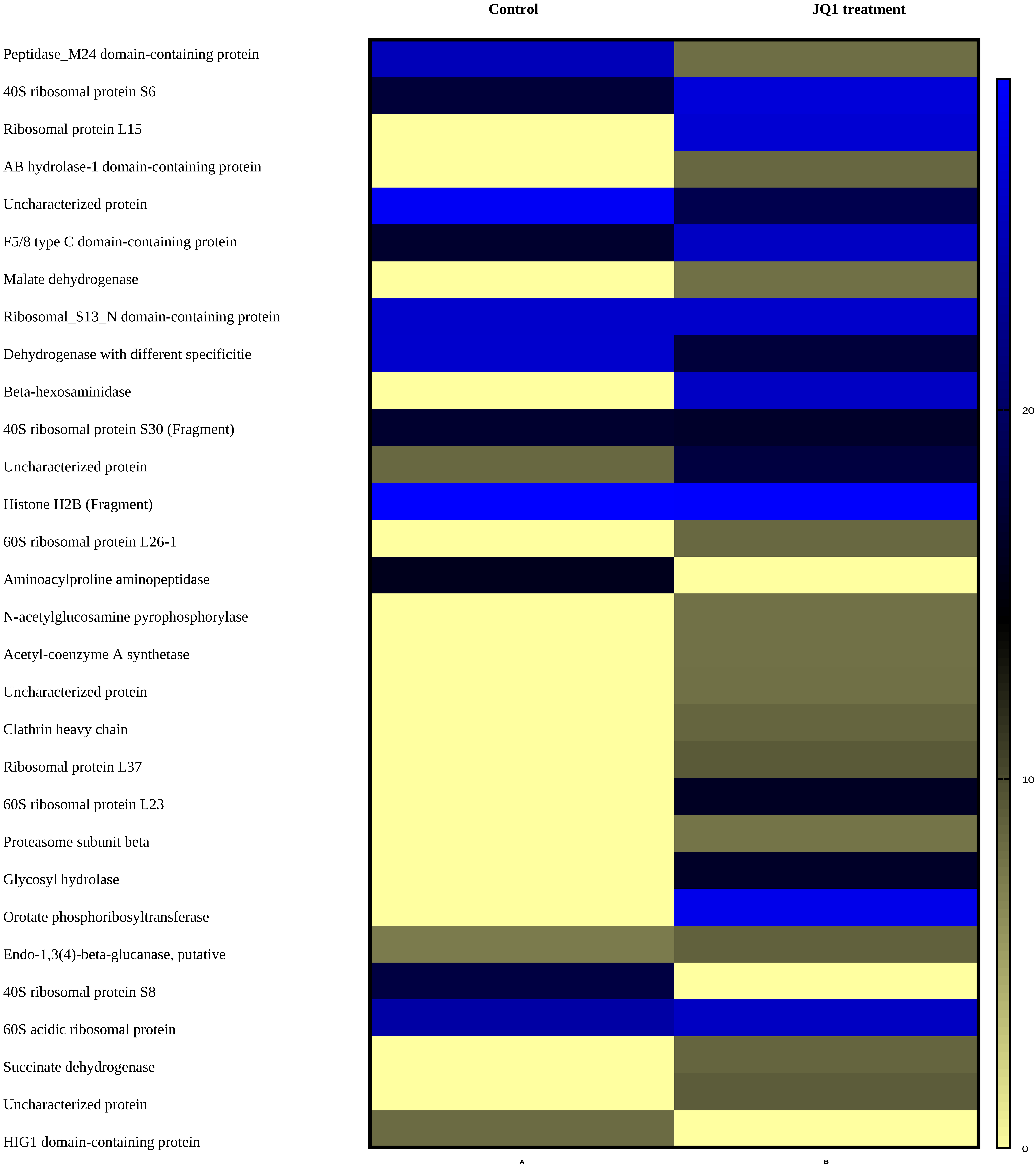

Supplementary S2. LC-MS/MS results of intracellular proteins by *A. fumigatus* DSM 790 after or not after JQ1 treatment. Overall data of LC-MS/MS analysis after treatment of *A. fumigatus* with or without JQ1. Log 2 (LFQ intensity) of the intracellular proteins. The value is expressed as media of at least three independent biological replicates.
